# Primary Pulmonary Leiomyosarcoma: A Case of Prolonged Survival With Radiotherapy and Pazopanib in a 90‐Year‐Old Female

**DOI:** 10.1002/rcr2.70301

**Published:** 2025-07-28

**Authors:** Sathish Krishnan, Vijaya Sivalingam Ramalingam, Chandrika Patel, Jennifer Heisick

**Affiliations:** ^1^ Division of Pulmonary and Critical Care Medicine Community Health Network Indianapolis Indiana USA; ^2^ Division of Pulmonary and Critical Care Medicine Northeast Georgia Medical Center Gainesville Georgia USA; ^3^ Division of Radiation Oncology Community Health Network Indianapolis Indiana USA

**Keywords:** leiomyosarcoma, lung cancer, palliative radiotherapy, pazopanib therapy, primary pulmonary leiomyosarcoma

## Abstract

Primary pulmonary leiomyosarcoma is an exceedingly rare malignancy, comprising less than 0.5% of all lung cancers. Due to its rarity, no standardised treatment guidelines exist. Prognosis is especially poor in cases which are deemed unresectable. We describe the case of a 90‐year‐old woman who presented with progressive dyspnea and cough over 1 month. Chest computed tomography revealed a large right hilar mass extending into the right upper and middle lobes, abutting the mediastinum and pericardium. Histopathological analysis confirmed primary pulmonary leiomyosarcoma. Given her advanced age and comorbidities, she was not a candidate for surgical intervention. Molecular profiling demonstrated PDL1 expression < 1% and no targetable mutations, ruling out the option of immunotherapy. She was managed with palliative radiotherapy followed by pazopanib therapy. Serial imaging demonstrated disease control, with an extended survival of approximately 5 years. Despite a median reported survival of 14 months, our patient achieved prolonged survival, highlighting the importance of an individualised therapeutic approach for elderly patients with rare malignancies.

## Graphical abstract

We present a case of an elderly female with unresectable primary pulmonary leiomyosarcoma who achieved prolonged survival with palliative radiotherapy and pazopanib therapy.

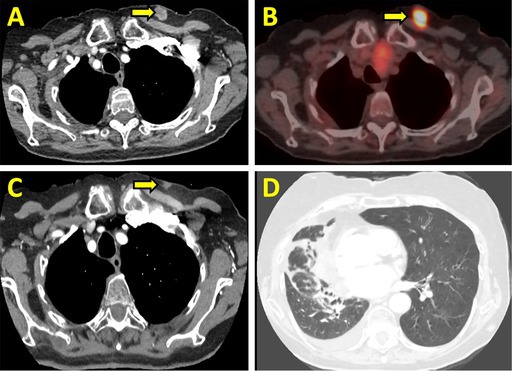

## 1 Introduction

Primary pulmonary leiomyosarcoma (PPL) is an exceptionally rare sarcoma arising from smooth muscle in the lung. Its incidence rate is reported between 0.2% and 0.5% among all pulmonary malignancies [1]. Due to its low prevalence, there is limited consensus on optimal management. Surgical resection remains the mainstay for localised disease. However, in cases of unresectable PPL, treatment options are limited [2]. We present a case of an elderly female with unresectable PPL who achieved prolonged survival with palliative radiotherapy and pazopanib therapy.

## 2 Case Report

**FIGURE 1:**
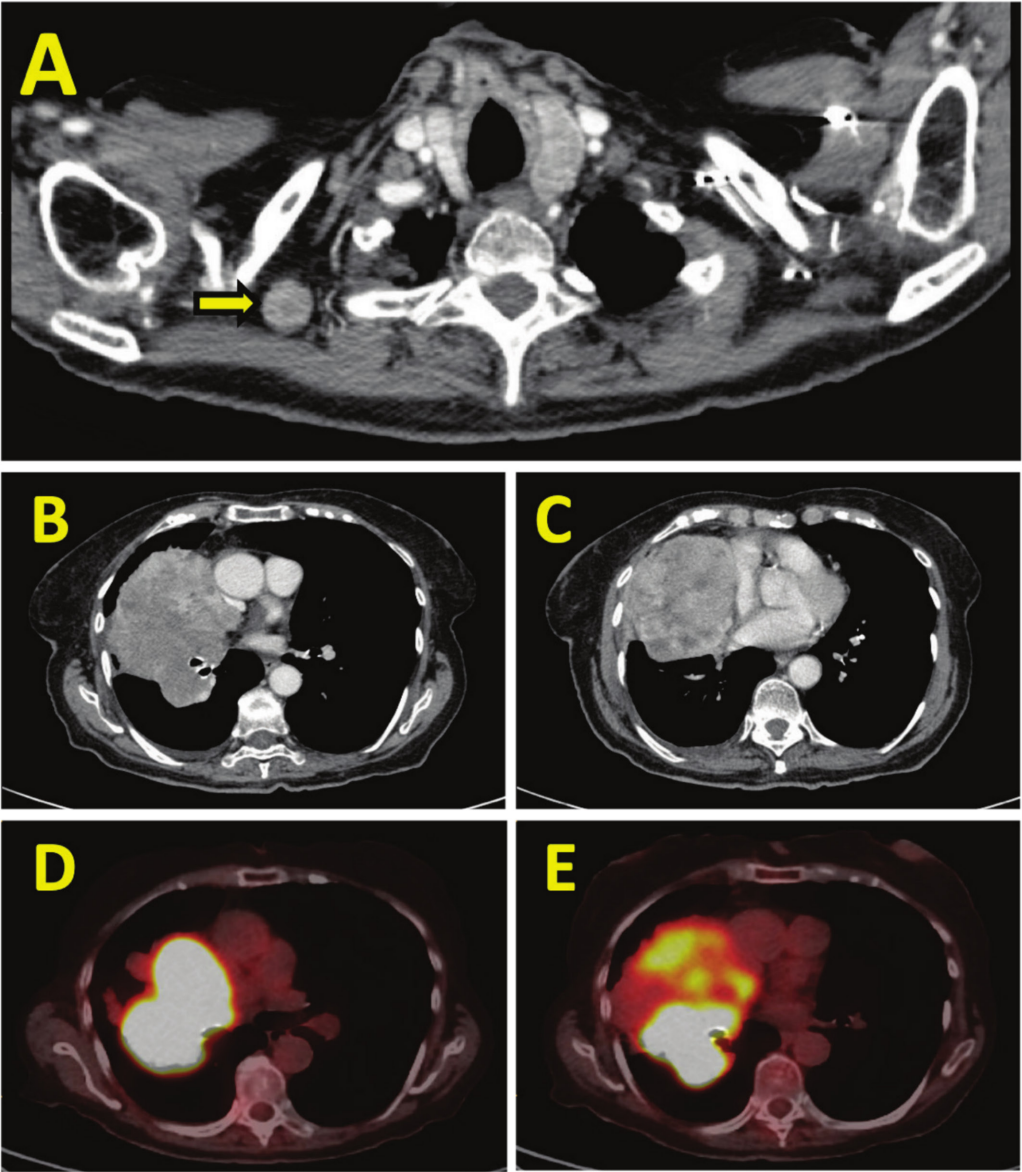
Axial CT chest images: (A) enlarged right supraclavicular lymph node (arrow); (B and C) right lung mass. FDG‐PET scan images: (D and E) demonstrate intense FDG uptake within the lung mass.

A 90‐year‐old female presented with a one‐month history of persistent cough and dyspnea. Her medical history was notable for hyperlipidemia, osteoporosis and gastroesophageal reflux disease. She was receiving treatment with alendronate, aspirin, atorvastatin, and omeprazole. Her Eastern Cooperative Oncology Group (ECOG) performance status was 2. Chest X‐ray demonstrated a large right hilar mass. Contrast‐enhanced computed tomography (CT) confirmed a 12 cm heterogeneously enhancing mass encompassing the right upper and middle lobes, abutting the mediastinum and pericardium, along with an enlarged right supraclavicular lymph node (Figure 1). Ultrasound‐guided biopsy of the supraclavicular node revealed an atypical smooth muscle tumour, suggestive of leiomyosarcoma, though a definitive diagnosis could not be established at that time. FDG‐PET scan exhibited intense metabolic uptake (SUV: 34) localised to the mass and no other areas of abnormal FDG uptake (Figure 1). MRI brain was negative for intracranial involvement. A subsequent percutaneous lung biopsy confirmed primary pulmonary leiomyosarcoma.

**FIGURE 2:**
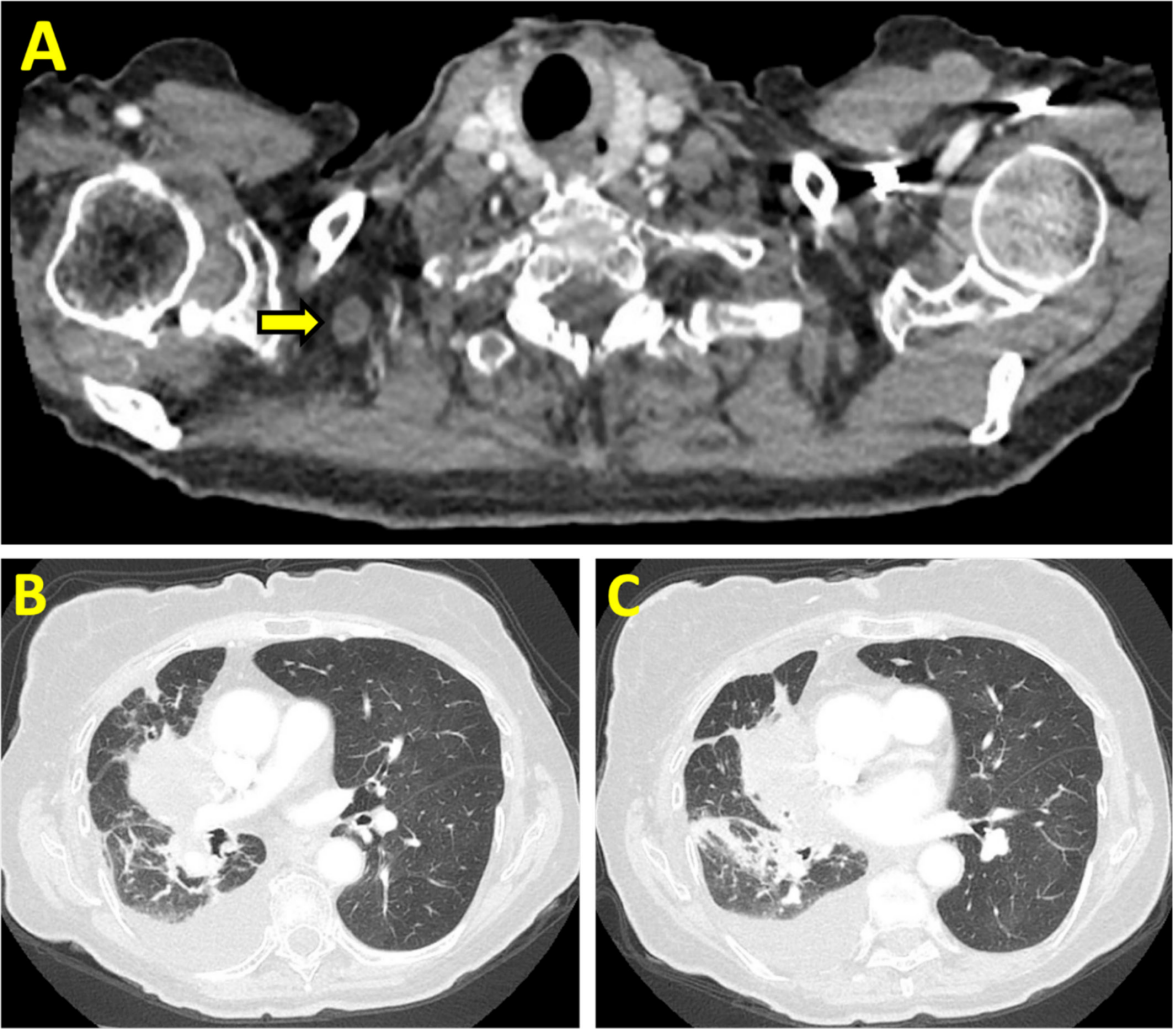
Axial CT chest images: (A) reduced size of right supraclavicular lymph node (arrow); (B and C) decrease in size of right lung mass.

Given her advanced age, she was ineligible for surgical resection. Her next‐generation sequencing (NGS) indicated PDL1 < 1% with no actionable mutations, which ruled out immunotherapy. So, she completed palliative radiotherapy (4500 cGy in 15 fractions over 4 weeks) targeting the right lung mass and supraclavicular node. Serial CT imaging at 3‐ and 6‐months post‐radiotherapy demonstrated significant tumour regression (Figure 2).

**FIGURE 3:**
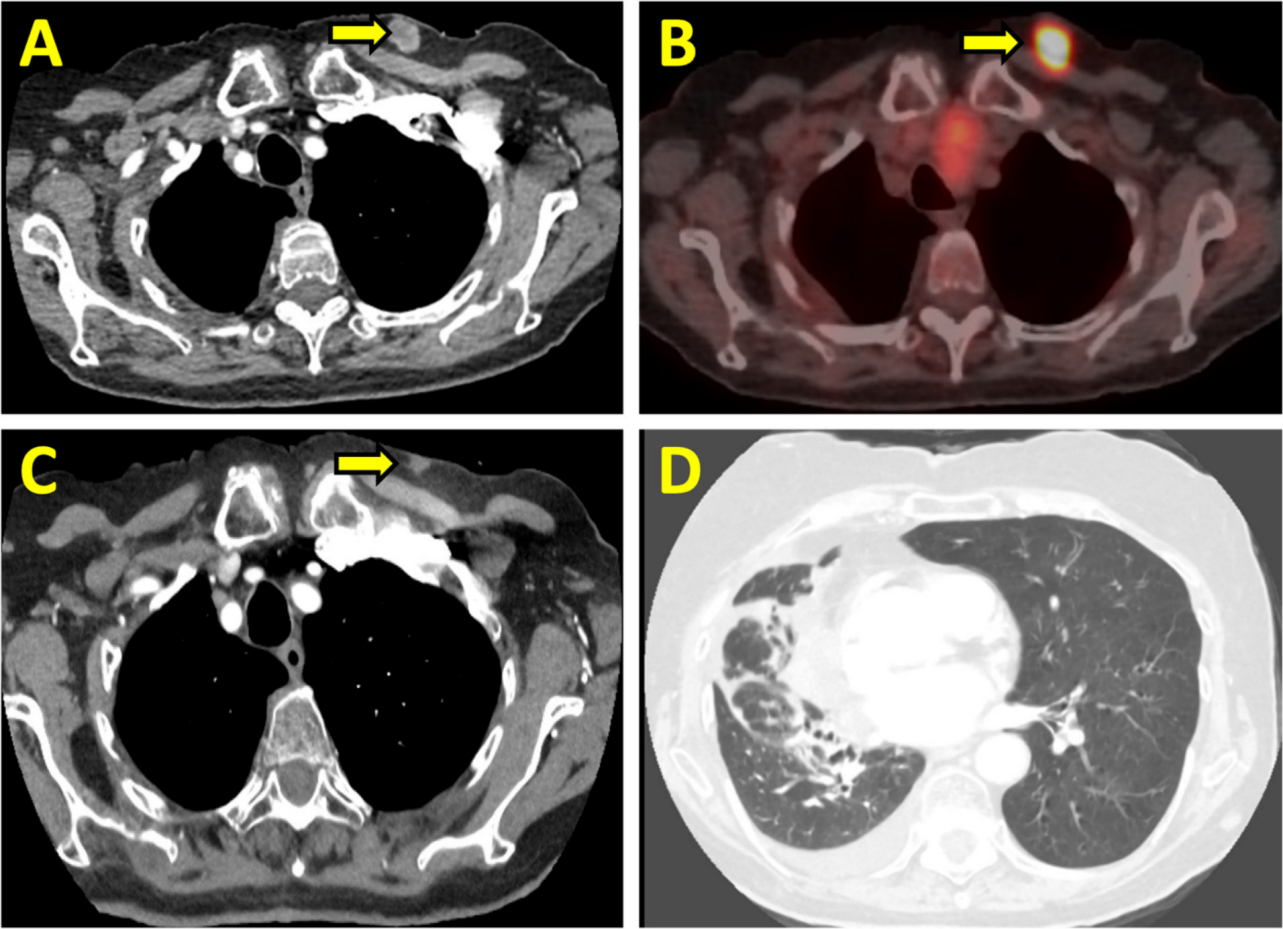
Axial CT chest and PET scan images: (A and B) new subcutaneous nodule in left upper chest wall with FDG uptake (arrows); (C) decrease in size of the subcutaneous nodule post radiotherapy (arrow); (D) progressive regression of right lung mass.

**FIGURE 4:**
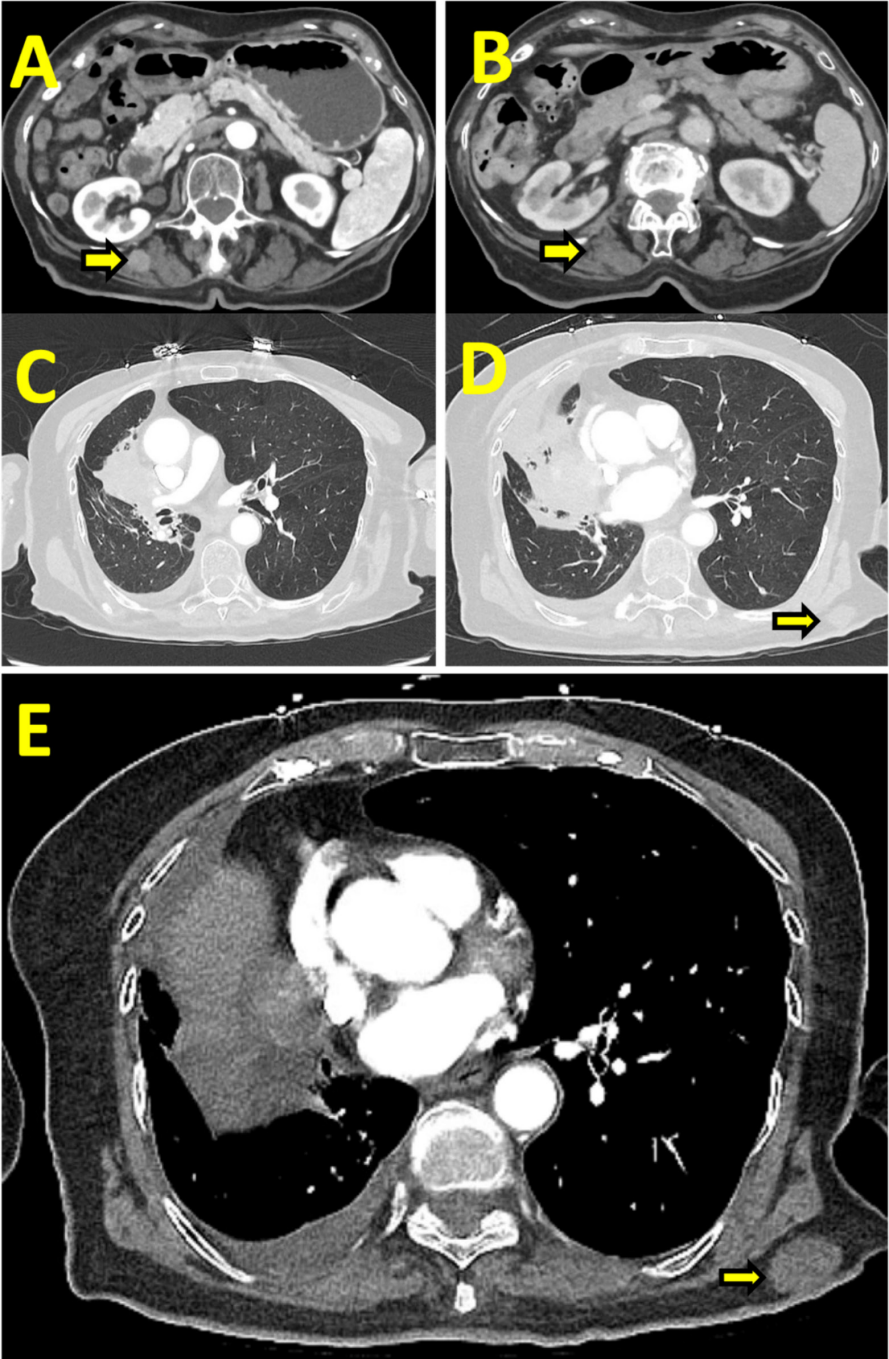
Axial CT chest images: (A) new soft tissue lesion between the right para‐spinal muscles (arrow); (B) decrease in size of the lesion post‐radiotherapy (arrow). (C and D) recurrence of right lung mass; (D and E) new subcutaneous lesion in left posterior chest wall (arrows).

Nine months post‐radiotherapy, surveillance imaging identified a new 1.7 cm subcutaneous left upper chest nodule, highly FDG‐avid on PET (Figure 3A,B). Biopsy confirmed metastatic leiomyosarcoma. She was started on pazopanib at 200 mg daily, escalating to 400 mg daily. Concurrently, she received an additional 4500 cGy (15 fractions) of palliative radiotherapy to the left chest wall. Three‐month follow‐up imaging showed a decrease in nodule size to 0.5 cm (Figure 3C,D). At 20 months, a 1.6 cm soft tissue nodule developed in the right paraspinal muscles (Figure 4A), and she completed additional palliative radiotherapy (3000 cGy in 15 fractions over one‐week). Follow‐up imaging confirmed tumour regression (Figure 4B).

The patient remained under surveillance for 3 years with no evidence of recurrence. At 61 months post‐diagnosis, she presented with syncope and her imaging revealed recurrent disease at the prior lung mass site with a new left posterior chest wall subcutaneous lesion (Figure 4C,D,E). She was also diagnosed with acute pulmonary embolism and left lower extremity deep vein thrombosis (DVT). Due to her declining functional status, she opted for hospice care.

## 3 Discussion

Primary pulmonary sarcomas are incredibly rare and arise from smooth muscle cells of pulmonary parenchyma, bronchi or pulmonary vessels. Based on the origin, PPL can be classified into three types: intraparenchymal, endobronchial and pulmonary vascular [3]. It accounts for less than 5% of all pulmonary malignancies, with leiomyosarcoma being the most predominant histologic subtype. Given the uncommon occurrence, the diagnosis can be challenging, often leading to delays in definitive treatment. Moreover, pulmonary leiomyosarcomas are mostly metastatic. Metastasis to the lungs occurs in about 40%–50% of patients with leiomyosarcoma. So, it is crucial to thoroughly evaluate for extrapulmonary sites of origin before confirming a diagnosis of primary pulmonary leiomyosarcoma [4].

The cornerstone of treatment is surgical resection, which is the only curative option. However, only one‐third of them are eligible for surgical resection due to the extent of tumour invasion [5]. Many patients present with advanced, unresectable disease, limiting treatment options to palliative approaches. A large‐scale population‐based study reported that the median overall survival rate was just 14 months and patients with advanced, unresectable disease had poorer prognosis [6]. Systemic therapies such as chemotherapy and targeted agents have limited efficacy due to the tumour's resistance to conventional regimens. Radiation therapy (RT) has been utilised for local tumour control, but long‐term survival remains poor [7]. Early studies in sarcomas primarily utilised anthracycline‐based regimens, with doxorubicin serving as the primary agent. More recent clinical trials have explored combination therapies, particularly doxorubicin with ifosfamide [8]. However, its role in primary pulmonary leiomyosarcoma is unexplored.

Pazopanib, a multi‐tyrosine kinase inhibitor, has shown modest efficacy in soft tissue sarcomas, though its impact on PPL remains unclear. It has demonstrated a significant benefit in extending median progression‐free survival in patients with metastatic soft tissue leiomyosarcoma [9]. A recent case report described tumour regression in a patient with PPL following 3 months of pazopanib therapy [10]. To the best of our knowledge, this is the first case in literature with long term follow‐up of a PPL patient treated with pazopanib. These findings suggest that pazopanib may offer a promising therapeutic option in the management of PPL. However, given the sequential nature of the treatment, it is difficult to precisely determine the extent to which pazopanib versus radiotherapy contributed to the observed clinical benefit. The relative impact of each modality remains uncertain and cannot be conclusively determined from a single case.

Our case has demonstrated the potential for prolonged survival in elderly patients with unresectable primary pulmonary leiomyosarcoma (PPL) through an individualised multimodal approach. Our patient, despite being a poor candidate for surgery, had 5 years of survival after diagnosis, which is significantly greater than the reported median survival for unresectable PPL. This implies that palliative radiotherapy along with targeted therapy may be absolutely important in controlling disease. The highly aggressive metabolic pattern of the tumour, indicated by high SUV on PET scans, generally has an unfavourable prognosis. However, the dramatic early response to radiotherapy favours its likely effectiveness in attaining local control even in nodal‐positive cases. The first metastatic lesion appeared approximately 9 months after diagnosis, while subsequent metastases emerged at one and 4 years following initiation of pazopanib. This temporal pattern suggests that the addition of pazopanib may have contributed to sustained disease stability. Although the precise contribution of each modality is difficult to delineate, the combination of radiotherapy and targeted therapy appears to have played a pivotal role in prolonging survival.

Throughout the initial workup and treatment course, the patient and her family actively engaged in in‐depth discussions with the multidisciplinary medical team to explore all available therapeutic options. A multidisciplinary care meeting was conducted prior to each round of radiotherapy to ensure that treatment decisions remained aligned with her evolving goals. Her preferences and comfort with the treatment plan were reassessed regularly. These discussions were grounded in the patient's deeply held values and priorities. During the early phase of treatment, when her ECOG performance status was 2, she chose to proceed with radiotherapy and pazopanib. As her condition declined and her performance status worsened to ECOG 3, she opted for hospice care. This transition reflected a commitment to respecting her autonomy and ensuring care remained consistent with her values.

In conclusion, primary pulmonary leiomyosarcoma (PPL) accounts for less than 0.5% of all pulmonary malignancies, making diagnosis and treatment highly challenging due to limited clinical experience and research. Since leiomyosarcoma more commonly presents as a metastatic disease to the lungs, establishing a primary pulmonary origin requires exclusion of extrapulmonary sources. FDG‐PET imaging plays a pivotal role in this diagnostic process by identifying or ruling out distant metastatic disease. In contrast to a historically unfavourable prognosis, our patient showed an extended survival of 5 years with palliative radiotherapy and pazopanib. Radiotherapy remains an effective tool even in unresectable cases for local control and in oligometastatic disease. Although the use of pazopanib in PPL is still being explored, this case illustrates the potential of pazopanib in maintaining disease stability and prolonging survival. As a result of the rarity of PPL, treatment algorithms are yet to be standardised, and further study into new combinations of treatment strategies is required.

## Author Contributions

**Sathish Krishnan:** conceptualization, supervision, writing – original draft. **Vijaya Sivalingam Ramalingam:** resources, writing – review and editing. **Chandrika Patel:** resources, visualization, writing – review and editing. **Jennifer Heisick:** resources, visualization, writing – review and editing.

## Ethics Statement

The authors attest that the submitted manuscript conforms to the ICMJE Recommendations for the Conduct, Reporting, Editing, and Publication of Scholarly Work in Medical Journals. Institutional Review Board approval not applicable given type of manuscript.

## Consent

The authors declare that written informed consent was obtained for the publication of this manuscript and accompanying images and attest that the form used to obtain consent from the patient's next of kin/healthcare power of attorney complies with the Journal requirements as outlined in the author guidelines.

## Conflicts of Interest

The authors declare no conflicts of interest.

## Data Availability

Data sharing is not applicable to this article as no new data were created or analyzed in this study.
